# Reasons and circumstances for the late notification of Acute Flaccid Paralysis (AFP) cases in health facilities in Luanda

**DOI:** 10.11604/pamj.2014.18.239.3770

**Published:** 2014-07-23

**Authors:** Arciolanda Macama, Joseph Okeibunor, Silvia Grando, Karim Djibaoui, Robert Koudounoaga Yameogo, Alda Morais, Alex Ntale Gasasira, Salla Mbaye, Richard Mihigo, Deo Nshimirimana

**Affiliations:** 1 World Health Organization, Angola; 2 World Health Organization Regional Office for Africa; 3 Ministry of Health, Angola

**Keywords:** Polio, acute flaccid paralysis, late notification, surveillance, Luanda, Angola

## Abstract

**Introduction:**

As the polio eradication effort enters the end game stage, surveillance for Acute Flaccid Paralysis in children becomes a pivotal tool. Thus given the gaps in AFP surveillance as identified in the cases of late notification, this study was designed to explore the reasons and circumstances responsible for late notification of AFP and collection of inadequate stools (more than 14 days of onset of paralysis until collection of the 2nd stool specimen) of AFP cases in health facilities equipped to manage AFP cases.

**Methods:**

Eleven AFP cases with inadequate stools were reported from January 2 to July 8, 2012 - Epidemiological Weeks 1-27. The families of these cases were interviewed with an in-depth interview guide. The staff of the seven health units, where they later reported, was also enlisted for the study which used in-depth interview guide in eliciting information from them.

**Results:**

Ignorance and wrong perception of the etiology of the cases as well as dissatisfaction with the health units as the major reasons for late reporting of AFP cases. The first port of call is usually alternative health care system such as traditional healers and spiritualists because the people hold the belief that the problem is spiritually induced. The few, who make it to health units, are faced with ill equipped rural health workers who wait for the arrival of more qualified staff, who may take days to do so.

**Conclusion:**

An understanding of the health seeking behavior of the population is germane to effective AFP surveillance. There is thus a need to tailor AFP surveillance to the health seeking behavior of the populations and expand it to community structures.

## Introduction

Angola interrupted transmission of indigenous wild polio virus and of imported virus which had re-established transmission. It has been polio free for more than a year, the last case of poliomyelitis due to transmission of indigenous wild poliovirus occurred on July 07, 2011. Despite the promising scenario, it is important to guarantee high quality acute flaccid paralysis surveillance, as there is a risk of importation of cases from areas where the disease is endemic [1]. Acute Flaccid Paralysis (AFP) refers to acute or sudden onset of weakness or paralysis of a limb characterized as flaccid (reduced tone) in a child < 15 years of age [2, 3]. AFP case was defined as a child aged less than 15 years showing acute onset of flaccid paralysis in one or more limbs, or acute onset of bulbar paralysis [4, 5]. The different forms of AFP diagnosis include paralytic poliomyelitis, Guillain-Barré syndrome and transverse myelitis; less common aetiologies are traumatic neuritis, encephalitis, meningitis and tumours. Distinguishing characteristics of paralytic polio are: asymmetric flaccid paralysis, fever at onset, rapid progression of paralysis, residual paralysis after 60 days, and preservation of sensory nerve function [5–7]. AFP surveillance is the prompt detection, investigation of flaccid paralysis of new onset in children under 15 years or any other suspected poliomyelitis case in a person of any age. Poliomyelitis (Polio) is a highly infectious disease caused by a virus. It attacks the nervous system, and can cause total paralysis in the infected person. It affects mainly children under three, though it can strike at any age [1]. One in 200 infections leads to irreversible paralysis [8]. Although polio-related paralysis is the most visible sign of polio infection, less than 1% of polio infections ever results in paralysis. Poliovirus can spread widely before cases of paralysis are seen. Given the silent nature of its transmission and the rapid spread of poliovirus, WHO considers a single confirmed case of polio paralysis to be evidence of an outbreak. Worse still, polio can only be prevented through immunization, since there is currently no cure for it. Adequate vaccination against polio almost always protects a child for life against AFP. All the same, surveillance is an essential tool in any campaign to eradicate disease and polio targeted for eradication in 2018, is no exception [9]. Only AFP cases with wild poliovirus in stool specimens are confirmed as polio, while those with adequate stool specimens that are negative for wild poliovirus are considered as non-polio cases. Adequate stool specimens are defined as two stool specimens (8-10 g) collected within 2 weeks after onset of illness, with an interval of more than 24 hours between collections, transportation on ice, and arrival at the laboratory in good condition (no desiccation, no leakage) [10]. This implies that early notification of all cases in health facilities is germane to a successful pursuit of the target of eradicating polio in Angola and ipso facto in the African Region. In Angola, where polio eradication efforts have recorded commendable success, stopping poliovirus transmission has been pursued through a combination of routine immunization and supplementary immunization campaigns which are guided by high quality AFP surveillance. However, late reporting of AFP cases has remained a major challenge. A recent survey of dates of notification of AFP cases in Luanda revealed that between the EPI weeks 1 and 27, a total of 42 AFP cases were reported. Of the total reported cases, 11 (26%) were reported with inadequate stools. It follows therefore that an understanding of the reasons and circumstance for late notification of AFP cases in the health facilities is a necessary first step to addressing the factors that militate against successful polio eradication initiative.

This paper results from a study designed to explore the factors responsible for the late notifications of AFP cases in the health facilities. The factors included individual perceptual and cognitive dispositions as well as cultural and structural issues. The effectiveness of disease eradication efforts, as is the case of all health interventions, not only relies on their clinical efficacy, but also on a range of factors, such as the perceptions, attitudes and behaviours of target groups, and of the wider community [11]. Attitudes and behaviours towards interventions are often shaped by social and cultural factors and such factors are particularly relevant to the response to the interventions. Attitudes towards and understandings of AFP and other illnesses can interact and influence how, where and when to respond. Furthermore, the social and cultural context has important implications for the uptake of polio immunization services, whether as part of routine health facility or during campaigns as is the case with even maternal health services in ANC or care sought from a local healer [12]. Data on knowledge and perceptions of AFP and the response to AFP cases like other health intervention, such as safe motherhood, have generally been collected using (more) quantitative methods, such as questionnaires, with comparatively little research based on (more) qualitative [13–16].

## Methods

### Study design and setting

The study employed the qualitative analytic methodology for a detailed analysis, interpretation and reflection of the data collected. It was conducted in Luanda Province. The focus was on the 11 cases with inadequate stools reported between week 1 and 27 in Luanda province. The cases are from the municipalities of Viana, Cazenga and Luanda (districts of Kilamba Kiaxi, Rangel and Sambizanga).

Luanda is the capital and largest city of Angola. Located on Angola′s coast with the Atlantic Ocean, Luanda is both Angola′s chief seaport and its administrative center. It has a metropolitan population of over 5 million. It is also the capital city of Luanda Province. A number of previously independent nuclei were incorporated into the city in 2011 making Luanda Province become divided into 7 municipalities. Table 1 gives a summary description of the Province with its internal distribution of population ( ≤5 years) and economic activities. The capital, Luanda is growing constantly and increasingly beyond the official city limits and even provincial boundaries, with many populations moving in from other areas. The inhabitants of Luanda are primarily members of African ethnic groups, mainly Ambundu, the Ovimbundu and the Bakongo. Around one-third of Angolans live in Luanda, 53% of whom live in poverty. Living conditions in Luanda are poor for most of the people, with essential services such as safe drinking water and electricity still in short supply, and severe shortcomings in traffic conditions. The population movement has implication for AFP surveillance and of course polio eradication initiative.

### Study population and sampling

The target population for this study is patients who were late in notification and subsequently develop AFP, as index persons. Their family members and the health facility staff covering the catchment area were enlisted in the study. A total of eleven families and 7 health professionals were purposively selected for the interviews.

### Instrument and method of data collection

The interviews were unstructured with a carefully developed interview guide, administered on both the families of the index persons and health workers. Interviews with health professionals from 7 health units that have not performed timely notification of AFP cases when patients sought care were conducted within the health units.

### Data analysis

Given the purpose of the study, which was to clearly elucidate the local perceptions of the presentation and progression of AFP experience, the analysis adopted a systematic and verifiable approach [16]. The process began by reviewing the interview experiences with the data collectors to obtain their views on factors that facilitated or hindered interaction with the study subjects. More detailed analysis began with reading of transcripts of the interviews. In going through the transcriptions, major concepts and phrases with contextual or special connotations were noted and pulled out as illustrative quotes. A second reading utilized a system of “open coding”, where broader illness processes were underlined using a colour scheme [17]. Next the symptoms and descriptions of AFP experiences were collated into a master list. The text was read again to discern patterns in the ordering and clustering of symptoms to learn about locally perceived presentation and progression of the infection or AFP case.

## Results

### Awareness of Acute Flaccid Paralysis (AFP)

The general idea of paralysis among the families of children with late case notification, who later became paralyzed, was laced in their notion of the disease etiology which is linked with supernatural causes. According to a member of the family of a child with late notification, paralysis is of various types, but these could be broadly classified by their cause. When asked if she was aware of the health condition referred to as acute flaccid paralysis, she retorted:

*Paralysis? Yes! I hear of paralysis at health units and vaccination campaigns. But I believe that is the one due to polio. The one my daughter has, is from traditional cause since she has completed her routine immunization schedule and has participated in all vaccination campaigns*.

This is typical of the nature of knowledge the people have of paralysis in their children. The study however revealed that even the health workers interviewed were not sufficiently knowledgeable on AFP. They blamed their poor knowledge on the level of training and refresher courses they have received. Three of the technicians interviewed were unable to decipher the technical meaning of the acronym AFP.

### Perception of Acute Flaccid Paralysis (AFP)

The interview at this point opened with an exploration of the perceptions the people hold about AFP with particular focus on their ideas of cause, symptoms among others. The general notion among the families of the cases with late notification was that AFP is a disease either caused by clinical complications associated with fever or traditional reasons having nothing to do with Polio or any known medical complications for that matter, especially where one has completed the vaccination schedule, According to one of the parents, *“My daughter had malaria first and later started showing difficulties on putting herself up even tough malaria she was already cured therefore I sought for a traditional physiotherapist”*. On the part of the health workers, AFP is a type of paralysis associated with high fever that demobilizes a febrile child. According to one of the health workers interviewed, *“Here at this Centre we have once assisted a similar case of paralysis the child ‘s neck was equally paralyzed and he cried a lot”*.

Further inquiry on the perceived causes of AFP in children showed mixed scenarios both among the parents and health workers. All the same, while the health workers blamed it on non-compliance with routine immunization regime, the notion of sorcery, jealousy and other non-medical causes came out strongly among the parents. The following quote stands typical of the dominant perceptions:

*It can either be of clinical causes due to noncompliance of immunization routines or traditional reasons, which is driven by jealousy. My husband ‘s second wife is infertile and put an evil eye on my daughter*.

Another mother said, *“My husband used to spend the night away from home; somehow he might have brought this disease to my son”*. On the other hand, one of the health workers interviewed noted that AFP is usually caused by *“Incompliance of the Routine Immunization Calendar, associated with irresponsible neglecting parents who don ‘t take their children to health units”*.

The symptoms, according to the health workers include fever, loss of limb muscle strength, diarrhea among others. The parents interviewed could not discuss the symptoms other than the sudden loss of limb muscle strength. One of the parents said, *“My daughter was doing well, was siting and suddenly fell off the chair and could no longer walk”. Another stressed that, “My son had no fever, just could no longer put his foot down”*.

### Health seeking for Acute Flaccid Paralysis (AFP) cases

AFP cases, if reported by the parents are managed at a central level within referral hospitals, National Vaccination Programme Centres, as well as Municipal Health Departments by the Public Health Epidemiologist responsible for endemic conditions. This is captured from a number of quotes from interviews with the health workers, list below:

*..We always transfer these cases to Health Centre or Hospitals of References, they do everything there* **(Respondent: IDI with a Nurse)**

*We call them from the municipal health department, there are doctors who know it is their responsibility..together with public health officials*.. **(Respondent: IDI with a Health Technologist)**

On the part of the families however, the first port of call is the traditional healer. This is because most of the parents believe the condition results from non-medical spiritual causes, better handled by equally spiritual traditional healers. The following quotes illustrate the pattern of health seeking among parents of children with AFP.

*.. I was there I paid everything they asked for, he gave her some teas and did her some massages but my daughter was not improving so we went to the hospital* **(Respondent: IDI with a parent of a late AFP notification case)**.

*..traditional healer asked us to pay 15,000 kwanza (equivalent 150.00$), while we were still collecting the money we took her to the nearest health post*.. **(Respondent: IDI with a parent of a late AFP notification case)**

With respect to the trajectory of response, the study revealed that families which tend to seek for help first at health units do so for reasons of economic constraints, accessibility and symptoms presented with the signs of AFP. Otherwise, many go for alternative medicine. Most of the families interviewed sought health care first at such alternative sources when AFP signs were observed. All the same, many engaged in “health-shopping”, a process of multiple and varied health seeking for diagnosis and treatment therapy, typical of poor patients, which often delays diagnosis and the start of treatment leading to more complications and outcomes [18]. It was also observed that most families are driven into “health-shopping” by discontentment with services provided by the traditional healer or even health units. The following quotes further illustrate the results.

*I was admitted for 2 days with my daughter sleeping in the same bed as three other babies and the doctors did nothing ..did not even give her some kind of medicine..we were kept waiting..and then they sent us to be transferred to another hospital and decided not to go and seek help at my village church*/ **(Respondent: IDI with a parent of a late AFP notification case)**

### Factors responsible for late case notification

Most families sought health in response to the paralysis late. Some waited until after the fourth day of the onset of the paralysis to take action. This is blamed on a number of factors ranging from ignorance on the parent of the families to the weakness of the health system. For most of the health workers to whom this inquiry was directed, the weakness in the system of AFP surveillance reverberates particularly as some of the health technicians who have the first contact with the patient, set the framework (diagnostics) and notifies the occurrence (outsourcing responsibility), often counsels the patient to go personally to the local unit of reference. So many cases escape from being caught or reported in due course. According to one of the health workers interviewed:

*We were counseled to personally transfer similar cases to hospital of references which have specialized staff to deal with these cases. We were not trained on how to deal with these particular complications option* **(Respondent: IDI with a parent of a late AFP notification case)**.

On the perceived relative effectiveness of the traditional health system compared to the orthodox medical system as a possible explanation of the late notification, both the health workers and families interviewed enumerated a number of proxy factors that explain the situation. The health workers stressed that AFP cases are most common at suburban areas, with low hygiene, low ranked economic and livelihoods standards. More so these areas have poor health infrastructures and poor health manpower providing urgent services to community members. Once an AFP case shows up at these facilities the health workers do not respond at these points, because they feel less capable to handle the case and tend to retain the case inside the facility until professional help arrives or immediately transfer the cases to a the local facility of reference. According to one of the health workers interviewed,

*..Whenever an AFP case shows up we usually call the public health epidemiologist.. Here we still do not have the capacity to handle these cases, so we always send all patients to the public pediatric hospital.. there they have trained doctors who will handle the situation much better*.

However, the families blamed it on inefficiency in the health units. According to a parent interviewed,

*For my son to have access to the provincial rehabilitation center we must carry with us the results of an X-ray sheet examination. I have scheduled that exam since May, always from a week to week I go back there only to find out that my day hasn ‘t arrived yet. My son is unable to walk straight up for 4 months now, what is there that I should do?*

Anxiety fueled by the people ‘s perception of the disease drive most families to seek help at the alternatives points. But it is also seen as an escape when public free services do not respond according to expectation (either inexistence or absence of professional aid or low standard medical assistance). In most cases they were frustrated, but they feel they have tried everything in their power. The evaluation is very aptly captured in the quote below:

*She began massaging sessions at the orthopedic Centre, twice a week and was stable, was already up but not really walking, after a month my cousin advised me to go seek the church..so we continue the massages in the church, the Pastor there was in charge of doing it and told us to go every day until August she was running again, and now she is well and good! Our high motivation was the fact that we could save the money spent in 3 taxis that we had to grab to reach the Centre*. **(Respondent: IDI with a parent of a late AFP notification case)**.

## Discussion

The study has confirmed a number challenges (ignorance, superstition to perceived and/or experienced inefficiency of the rural or sub-urban health systems) faced by the people in managing AFP cases and hence late notification of cases. range from. Generally, alternative health systems like traditional physiotherapy, traditional healer and spiritual churches come as primary options while the health system is used by those faced with economic challenges because treatment in the health services are free. Results from this study indicated that most of the families delayed in seeking appropriate care because of the absence of efficient health service in the communities. In many cases, the families waited for the arrival of professionals from the city in the midst of great anxiety. It becomes evident that epidemiological surveillance needs to be implemented at community and health unit bases rather than the current practice which is more at health structures levels. A direct contact with community alternative care providers is made necessary. This would facilitate the early detection and notification of AFP cases. This study revealed some levels of awareness of AFP in the communities. All the same, the level of awareness did not correspond with actual practice on case AFP case notification among the families. Earlier studies on health seeking behavior have demonstrated that behavior is poor not because of lack of knowledge of improved technologies but because of a lack of demand due to prevailing perceptions about the new technologies [19]. In this study, we found that a number of factors constrain the families from adequate AFP case notification. These factors are aptly captured in the health belief models (HBM), focusing on the attitudes and beliefs of individuals which help predict or explain health behaviours and practices [20].

The threat-efficacy model [21] describes the process of people being exposed to a threat, assessing their level of susceptibility to the threat and its perceived severity. The model holds that when someone perceives some severe threats to health, s/he first assesses his or her own ability to manage the threat. If the individual feels that s/he knows of an effective strategy to deal with the threat and is confident of being able to implement this strategy, the person is said to have high perceived efficacy and will initiate a danger control response to mitigate the threat. On the other hand, perception of a low self-efficacy to address the threat will compel the individual to initiate a “fear control response” which will not address the threat itself, but rather will deny or ignore the threat. It follows therefore that threat inherent in parents seeing their child paralyzed is sufficient drive to seek the faster remedy perceived to be effective rather than wait on the health system.

Some of these barriers have been recognized in such other research undertaken in other climes and relating to health seeking generally and infectious disease control like tuberculosis control in particular [22–24]. Research to understand people ‘s knowledge, beliefs, and practices with regard to managing infectious diseases has the potential to lead to improvements in patient compliance with treatment [24], but more importantly to improve community access and use of clinics specially designed to help with the management AFP cases is adequately notified. The participants in this study identified a number of drivers of late notifications and primary among which is the erroneous belief that this is caused by envious or wicked neighbours. Other reasons include the slow response of the health workers, when they do. In many cases the health workers lack the capability to handle the cases.

The weakness in the system of surveillance of AFP reflects particularly on how the family approaches the physical complication (place where first aid is sought) as well as on how the health technician who has the first contact with the patient, sets the framework (diagnostics) and notifies the occurrence (outsourcing of responsibility) often orientating the patient to personally go to the reference unit. Structuring the health workers as an actor of epidemiological surveillance network is a major challenge.

## Conclusion

It is necessary to holistically implement epidemiological surveillance in order to facilitate early detection of AFP cases. Production of tools for health promotion and community education directed at leaders who provide health services in the community should be considered. The intention would be to sensitize them on the importance of monitoring, detection and reporting AFP suspected polio cases. Those activities could be coordinated with the community ‘s closest health unit, and health activists. Health care workers should be trained to handle APF suspected cases as they come rather than simply applying the notification methodology only and awaiting the arrival of the technical team. The logistics of communication structures for reporting and technical guidance as well as transportation of stools, control and delivery at laboratory must be strengthened for the sake of the feasibility of the surveillance methodology.
